# Coffee-ring effect enables correlative super-resolution imaging of label-free chromosomes

**DOI:** 10.1098/rsif.2026.0072

**Published:** 2026-10-14

**Authors:** Yanxin Dou, Valerie S. T. Goh, Thomas Osipowicz, Christelle E. L. Chua

**Affiliations:** ^1^ Institute of Modern Physics, Chinese Academy of Sciences, Lanzhou, People's Republic of China; ^2^ Singapore Nuclear Research and Safety Institute, National University of Singapore, Singapore; ^3^ Centre for Ion Beam Applications, Department of Physics, National University of Singapore, Singapore

**Keywords:** correlative imaging, super-resolution, label-free, chromosome, coffee-ring effect

## Abstract

High-resolution chromosome imaging plays a key role in understanding chromosome structure. However, traditional methods for super-resolution (<200 nm) chromosome imaging are often complex and time-consuming, requiring purification, staining or sectioning of chromosomes. Here, we propose a novel and simple method to achieve correlative super-resolution imaging for label-free chromosomes through the utilization of the coffee-ring effect. An application of this method is shown to identify dicentric chromosomes and acentric fragments.

## 1. Introduction

The 300–700 nm width range of human chromatid arms demands super-resolution (<200 nm) microscopy for structural resolution [1–3], as conventional methods lack sufficient resolving power. Label-free [4] and correlative microscopy [5,6] are also highly desirable in chromosome imaging. However, traditional methods for correlative super-resolution imaging of label-free chromosomes are often complex and time-consuming, requiring purification, staining or sectioning of chromosomes [7]. Here, we developed a novel chromosome isolation technique based on the coffee-ring effect to simplify chromosome analysis procedures. The coffee-ring effect is a phenomenon in which non-volatile solutes are deposited in ring-like fashion when a drop of liquid dries on a solid surface [8,9]. The non-volatile solute deposition process is influenced by a number of aspects such as the solvent drying conditions (including substrate temperature, relative humidity and solvent volatility) [10], particle shape [9,11] and surface charge of the substrate and particle surfaces [12]. We demonstrated that the coffee-ring effect enables chromosome isolation, thereby enabling the development of a label-free correlative super-resolution imaging technique for chromosomes. Specifically, the coffee-ring effect allowed the deposition of isolated chromosomes on a silicon (Si) wafer or an ultra-thin carbon film. Consequently, the isolation of chromosomes on these substrates facilitated high-resolution chromosome imaging without chemical staining or physical sectioning, enabling seamless integration of transmission electron microscopy (TEM), nuclear microscopy (NM), scanning electron microscopy (SEM) and atomic force microscopy (AFM).

This label-free correlative super-resolution imaging approach facilitated the identification of unstained dicentric chromosomes, a type of radiation-induced chromosome aberration. A dicentric chromosome contains two centromeres, in contrast to a single centromere found in a normal chromosome. Dicentric chromosomes are typically analysed in the dicentric chromosome assay, which is internationally recognized as the ‘gold standard’ of biological dose assessment in suspected exposures of radiation accidents [13,14]. Beyond dicentrics, this label-free correlative super-resolution imaging approach shows great potential for studying a wider array of subtle chromosome structures and aberrations, opening new avenues in fundamental chromosome biology and cytogenetics.

## 2. Material and methods

### 2.1. Normal chromosome preparation

Peripheral blood from healthy donors was collected in sodium heparin blood collection tubes (Greiner Bio-One) with their informed consent. Donor recruitment was approved by the National University of Singapore Institutional Review Board (H-17-068).

Peripheral blood mononuclear cells (PBMCs) were isolated from blood via density centrifugation using Ficoll-Paque (Cytiva) and SepMate-15 tubes (STEMCELL Technologies), following the manufacturers′ protocols. PBMCs equivalent to 1 ml of whole blood were cultured in 5 ml of RPMI-1640 medium (Thermo Fisher Scientific). The culture medium was supplemented with 20% heat-inactivated foetal bovine serum (Cytiva), 1× penicillin/streptomycin (Thermo Fisher Scientific), and 2% phytohemagglutinin (Thermo Fisher Scientific). Cells were incubated at 37°C in a humidified atmosphere with 5% CO₂. A total of 0.05 µg ml^−1^ colcemid (Thermo Fisher Scientific) was added 24 hours after culture initiation to induce metaphase arrest.

After 48 hours, cells were harvested and fixed. After the first round of centrifugation (400 × *g*, for 5 minutes, at 4°C), cold 75 mM potassium chloride (KCl) was added for hypotonic treatment for 20 minutes. Cold fixative (methanol : acetic acid, 3 : 1) was added, and cells were incubated for 5 minutes at 4°C. After centrifugation, cells underwent three rounds of fixation in cold fresh fixative. Finally, cells were resuspended at appropriate concentrations in the fixative and stored at −20°C.

### 2.2. Dicentric chromosome preparation (optional)

The procedures for dicentric chromosome experiments followed those for normal chromosomes described above, with the addition of an X-ray irradiation step. Specifically, before PBMC isolation, 1 ml blood aliquots in 15 ml conical centrifuge tubes were irradiated with 3 Gy X-rays (Xrad-320, Precision X-ray) and subsequently incubated at 37°C for 2 hours to allow for deoxyribonucleic acid (DNA) repair.

### 2.3. Chromosome isolation

Substrates were preheated at 60°C for 10 minutes on a hotplate. Room temperature fixed chromosome suspensions were dropped on substrates using a micropipette, followed by 10-minute air-drying. A carbon film substrate is a 3- to 4-nm-thick carbon film deposited on a TEM grid (Electron Microscopy Sciences CFLF135-Cu-UL). A Si substrate is a cut section of Si wafer (WaferPro C04001, 525 µm thickness, single-side polished). Droplet volumes of fixed chromosome suspensions were 0.3 µl for carbon substrates and 0.5 µl for Si substrates.

### 2.4. Chromosome staining (optional)

After chromosome isolation, air-dried chromosomes on Si substrates were stained for 10 minutes in the dark with Hoechst 33258 (Thermo Fisher Scientific) diluted 1 : 1000 in phosphate-buffered saline (PBS), followed by a PBS wash. This is in accordance with manufacturer's protocol.

### 2.5. Imaging methods

Optical microscopes (Olympus CKX53, Nikon Eclipse LV100ND) were used to check the chromosome spreading and chromosome isolation. Imaging of Hoechst-stained chromosomes was performed with a laser scanning microscope (Zeiss LSM 880) at the manufacturer's recommended wavelength with excitation at 350 nm and emission at 460 nm for Hoechst 33258.

High-resolution imaging tools are TEM, NM, SEM and AFM. The TEM (JEOL JEM-3011) operated at an accelerating voltage of 300 kV in a bright-field mode. Developed in-house, the NM is the world's first nuclear microscope achieving sub-10 nm resolution [15]. In this work, the NM operated in an off-axis scanning transmission ion microscopy (off-axis STIM) mode using 1.5 MeV hydrogen molecular ions (H_₂_⁺). The SEM (JSM-6700F) was conducted at an accelerating voltage of 1.0 kV to acquire secondary electron signals. The AFM (Bruker Dimension ICON) was performed in tapping mode to obtain height signal data.

## 3. Results

### 3.1. Correlative super-resolution imaging for label-free chromosomes

Figure 1 shows correlative super-resolution imaging for label-free chromosomes. Four super-resolution imaging technologies (TEM, NM, SEM and NM) were used to present different signals from chromosomes. The procedures are shown in figure 1A, including chromosome preparation, chromosome isolation and imaging. A novel chromosome isolation method was developed based on the coffee-ring effect, simplifying the chromosome isolation procedure.

**Figure 1.**
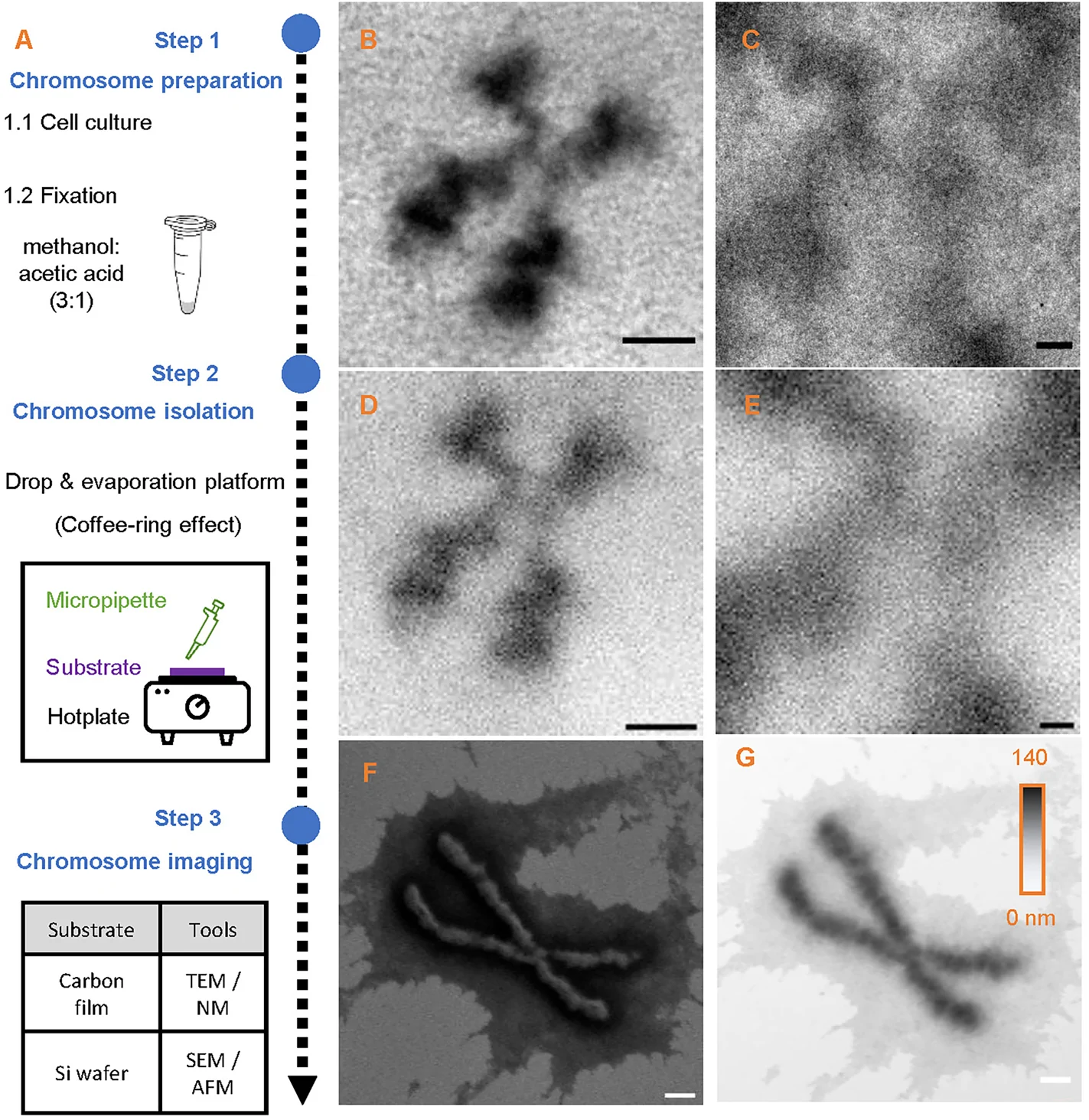
Correlative super-resolution imaging for label-free chromosomes. (A) Schematic illustration of chromosome analysis procedures. Chromosome imaging by (B,C) TEM, (D,E) NM, (F) SEM, and (G) AFM. The height scale in (G) is 140 nm. TEM and NM jointly imaged one chromosome on carbon film, whereas SEM and AFM characterized another chromosome on a silicon (Si) wafer. Scale bars, (B,D,F,G) 1 µm and (C,E) 200 nm.

A representative chromosome on a carbon film substrate was characterized by correlative TEM (figure 1B,C) and NM (figure 1D,E), revealing consistent structural features. Sub-200 nm resolution was achieved in centromere imaging by both TEM and NM (figure 1C,E), confirming high-detail visualization capability. More experimental data for NM resolution measurements can be found in the supplementary material, figures S1–S3. Correlative SEM (figure 1F) and AFM (figure 1G) characterization of a representative chromosome on a Si substrate revealed consistent structural morphology. The electron-induced signal strength was quite different in different areas of the chromosome (figure 1F). The thickness of air-dried chromosome was less than 140 nm (figure 1G).

### 3.2. Chromosome isolation using the coffee-ring effect

A widely used method for chromosome imaging on glass slides relies on methanol : acetic acid fixation, which preserves chromosomal structure and promotes cell lysis upon drying, yielding a monolayer spread [16,17]. In this basic technique, no physical separation between chromosomes and cell debris occurs; this poses little issue for conventional low‑resolution microscopy, especially with staining. However, such substrates and protocols are incompatible with super‑resolution label‑free imaging, which requires both suitable surfaces and a clean background. We therefore turned to the coffee‑ring effect, the principle of which is depicted in figure 2A. During droplet evaporation, solid particles are sorted by size at the pinned edge [12,18]. Because chromosomes are much larger than other particulate species present in the fixed cell droplet—including cell debris and reagent‑borne impurities—we reasoned that this effect could be harnessed to physically isolate chromosomes on super‑resolution‑compatible supports.

**Figure 2.**
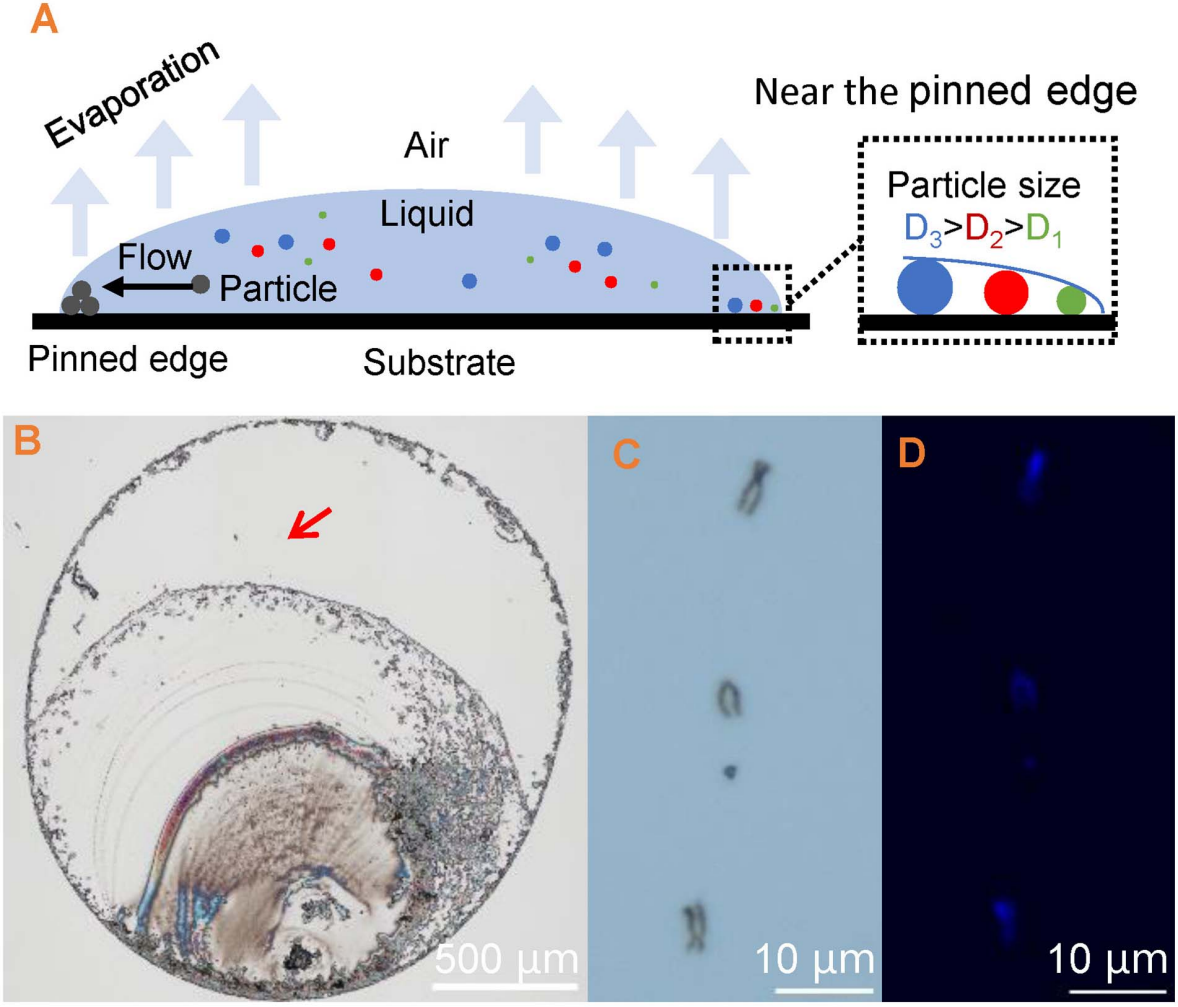
Chromosome isolation using the coffee-ring effect on a Si substrate. (A) Schematic diagram of the coffee-ring effect and its particle separation capability. (B,C) Optical images. (D) Confocal image. The area highlighted with a red arrow in (B) is magnified in (C,D).

As shown in figure 2B, after the sample containing chromosomes was air-dried on the Si wafer, a ring-shaped pattern was formed and the ring-shaped deposition bands showed the spatial separation characteristics of different solid particles. Owing to the significant differences in geometric morphology between chromosomes and other solid particles, chromosomes could be directly identified based on their distinct phenotype, as shown in figure 2C.

To confirm that the solid substances separated based on geometric shapes were indeed chromosomes, Hoechst 33258 was used to stain for DNA material found in chromosomes. Fluorescence signals of Hoechst 33258 on the chromosomes were observed with confocal microscopy imaging (figure 2D), verifying the effectiveness of chromosome recognition based on morphology. However, the fluorescence pattern in figure 2D failed to clearly show the geometric shape of the chromosome, and there were significant differences in the fluorescence intensities of different chromosomes and even different regions of the same chromosome. Because there is no expectation of any fluorescence quenching as the chromosomes are cell-free and the fixative used is not known to quench Hoechst 33258, it is probable that the inconsistency is owing to the non-uniform nature of the label. This indicated that the fluorescence labelling method had limitations: it was difficult to achieve uniform labelling across the entire chromosome.

The coffee-ring effect, as a physical effect, theoretically has basal universality. We confirmed that chromosome isolation could also be achieved on the carbon membrane substrate (figure 3). It is worth noting that the drying parameters need to be optimized for different substrates in order to effectively utilize this effect for chromosome isolation. We further demonstrated that the coffee‑ring effect can be applied to isolate chromosomes from other cell types, such as Chinese hamster ovary (CHO) cells (supplementary material, figure S4).

**Figure 3.**
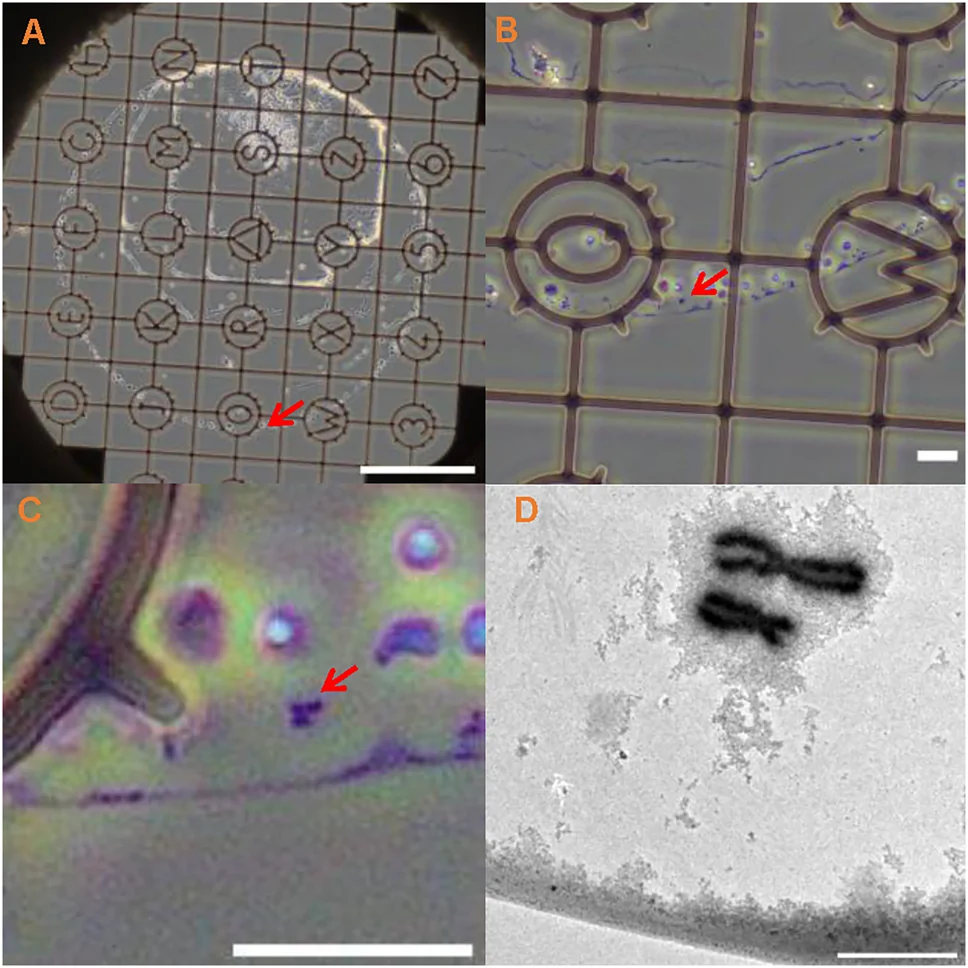
Chromosome isolation using the coffee-ring effect on a carbon film substrate. (A–C) Optical images. (D) TEM image, which is a magnified view in (A–C), indicated by a red arrow. Scale bars, (A) 500 µm, (B,C) 50 µm and (D) 5 µm.

There are various methods to control the coffee-ring effect to change the distribution of solid substances, and regulating the evaporation rate of liquids is one of them. In this study, the evaporation rate of the liquid was controlled by changing the substrate temperature, thereby regulating the distribution of solid substances (figure 4). The experiment found that when the substrate temperature exceeded 30°C, the solid substances in the sample formed multiple distinct separated regions. This indicated that temperature regulation during the chromosome spreading process could be used for chromosome isolation. For figures 1–3 and 5, we maintained the substrate temperature at 60°C. Normally, more than five well-shaped chromosomes could be obtained per droplet (0.3 µl) in our work.

**Figure 4.**
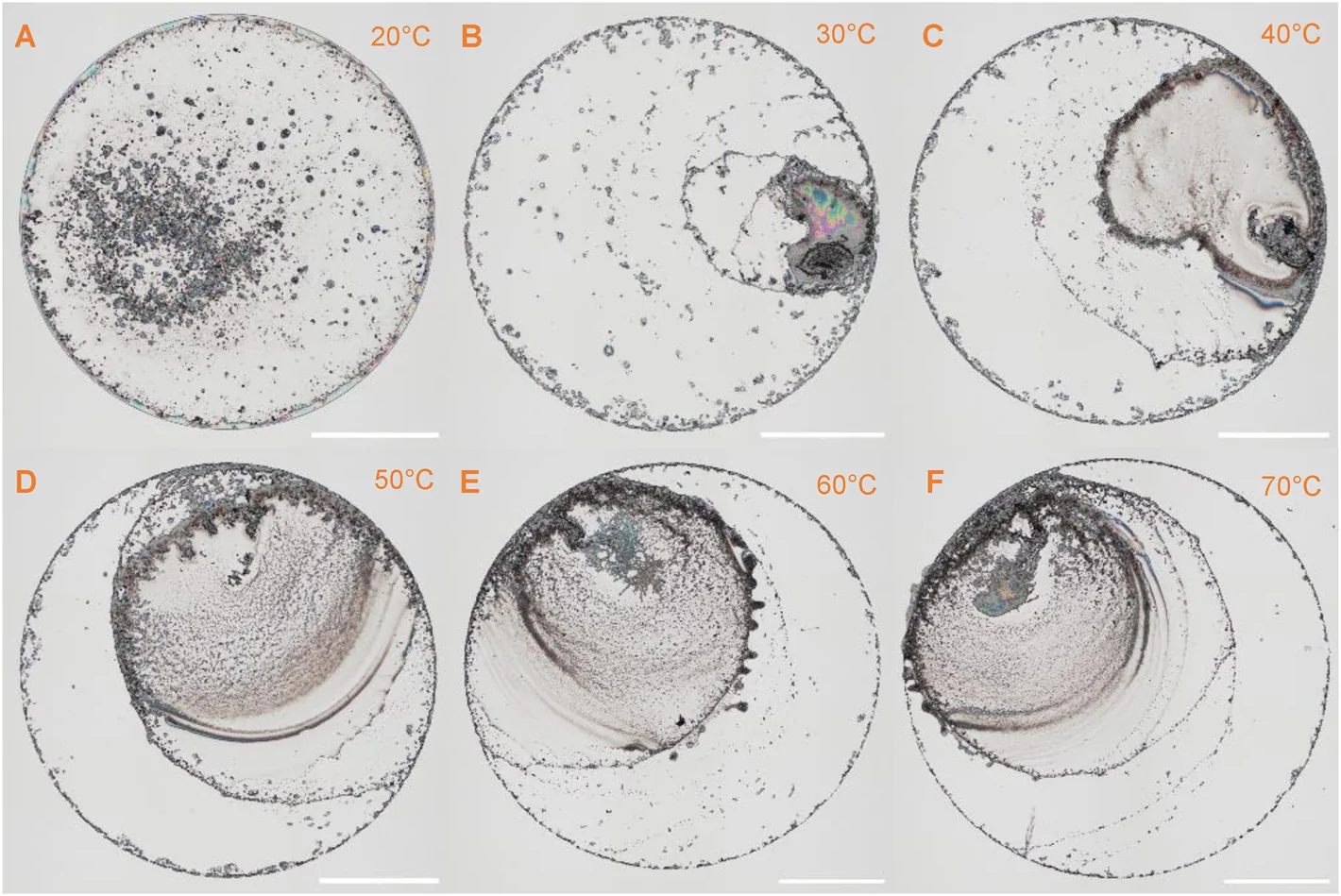
Controlling the coffee-ring effect through temperature variation. Images were taken by an optical microscope. Scale bar, 500 µm.

**Figure 5.**
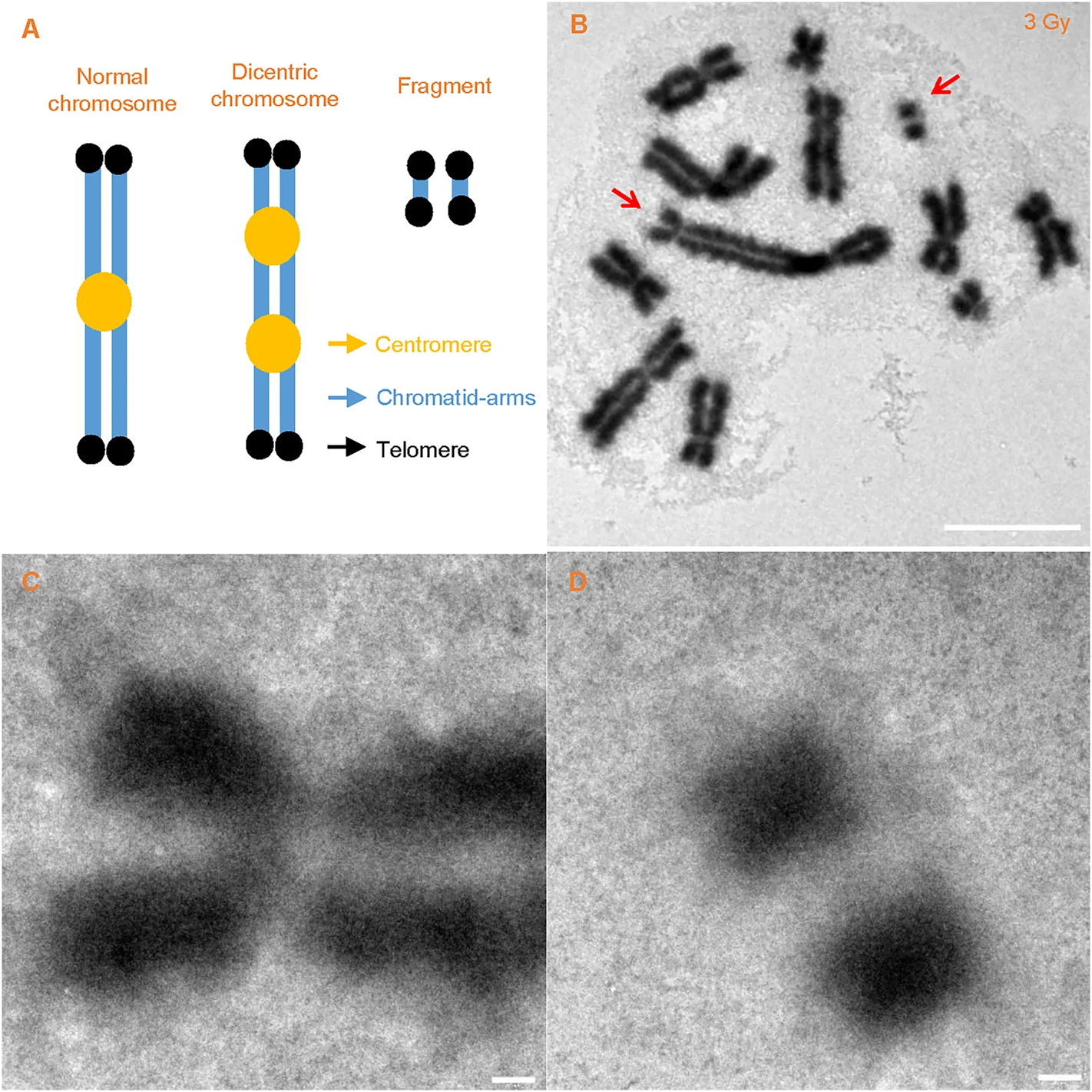
TEM images of normal and dicentric chromosomes. Dicentric chromosomes were formed after X-ray irradiation. (A) Schematic diagram of normal and dicentric chromosomes and fragments. (B–D) TEM images. (C,D) Magnified views in (B), indicated by red arrows. Scale bars, (B) 5 µm and (C,D) 200 nm.

### 3.3. Dicentric chromosome identification

A new imaging method constructed based on the coffee-ring effect was applied to the precise identification of dicentric chromosomes as an example of the possibilities enabled by the technique. Figure 5 shows the imaging results of one of the centromeres of a dicentric chromosome and its fragments. Figure 5A is a schematic diagram of the structure of normal and dicentric chromosomes, and dicentric chromosomes are usually produced along with fragments. TEM could clearly distinguish the centromeres of dicentric chromosomes (figure 5B,C) and clearly image fragments smaller than 500 nm (figure 5D), effectively ruling out the possibility that the morphology of double centromeres was caused by the close adjacency or superposition of two normal chromosomes. More TEM images of normal and dicentric chromosomes can be found in the supplementary material, figure S5.

## 4. Discussion

Although traditional metaphase chromosome spreading on glass slides is commonly performed for chromosome studies [16,19], the thickness and surface roughness of glass slides hinder its application in high-resolution electron or ion microscopy. Carbon films are widely used in electron microscopy for their low imaging interference and good electrical conductivity. The softness of carbon films limits their use in AFM, making Si wafers a more viable substrate for AFM imaging. Therefore, both carbon films and Si wafers are explored in this paper to use TEM, NM, SEM and AFM. Similar chromosome spreading characteristics were observed on carbon films, Si wafers and glass substrates; humidity is the most important variable affecting the quality of chromosome spreads [16]. However, the metaphase spreading process on slides is frequently hindered by cytoskeletal contaminants and residual chemical interference. The combination of sucrose and density gradients method isolates pure metaphase chromosomes from human cells [20], though its operational complexity limits widespread adoption. Alternative approaches for preparing chromosome samples for label-free super-resolution microscopy—including cryo-vitrification [21] and resin embedding [22]—involve technically demanding protocols.

This paper proposes a novel and simple method for chromosome isolation based on the coffee-ring effect, providing the possibility for the combined use of multiple high-resolution imaging tools to achieve label-free chromosome imaging. Label-free imaging has extremely stringent requirements for the purity of chromosome samples. Impurities can result in high background, which significantly interferes with the accurate analysis of chromosome structures. Our method provides a way to obtain isolated chromosomes with minimal interference from impurities.

Theoretically, there are numerous factors influencing the coffee-ring effect, such as the shape of solid particles, air humidity, temperature, substrate friction coefficient, liquid volatility etc. [10,12]. For this reason, theoretically, there are multiple ways to manipulate chromosome distribution by regulating the coffee-ring effect. Among them, changing the substrate temperature and fixing others is a simple and practical strategy. For successful chromosome isolation, other conditions must be optimized and fixed to make the particle-size selection mechanism in the coffee-ring effect work. This study confirmed that an optimally selected substrate temperature can be used for chromosome isolation based on the coffee-ring effect.

Furthermore, the combination of multiple imaging techniques can aid in effectively eliminating the interference of artefacts. This paper presents two combination strategies: one is to achieve the combination of TEM and NM on a carbon film substrate, and the other is to achieve the combination of SEM and AFM on a Si wafer substrate. TEM is suitable for high-resolution analysis of thin samples, whereas NM is suitable for high-resolution analysis of thick samples. The two effectively complement each other in terms of sample thickness adaptability. AFM can directly provide information about chromosome thickness, whereas SEM mainly reflects the differences in secondary electron yield in different regions of chromosomes. Surface roughness of Si wafers (<1 nm) could be ignored during AFM measurement of human chromosome thickness (>50 nm). Theoretically, the development of adaptive substrate materials is expected to achieve the integration of multimodal imaging of TEM, NM, SEM and AFM, thereby greatly expanding the dimension of chromosome structure research. This integration strategy will become the key direction of our subsequent research.

## 5. Conclusions and outlook

This study demonstrates efficient chromosome isolation by exploiting the coffee-ring effect, enabling label‑free characterization of individual chromosomes using multiple high‑resolution imaging tools. We show that the deposition behaviour can be rationally controlled by adjusting the substrate temperature, thereby improving the isolation efficiency. Furthermore, the method is broadly adaptable to various substrates, including carbon films and Si wafers, and proves effective for visualizing individual chromosomes and identifying structural aberrations such as dicentric chromosomes.

Looking forward, this technique holds considerable promise for advancing high‑resolution microscopy in cytogenetics and beyond. For instance, extending the coffee‑ring phenomenon to fixatives other than methanol : acetic acid, or to different colloidal systems, may unlock compatibility with a wider range of analytical techniques, such as scattering‑type scanning near‑field optical microscopy, photoinduced force microscopy or X-ray imaging. More broadly, the simplicity and substrate flexibility of our approach suggest its potential for integration into routine sample preparation workflows, potentially facilitating label‑free, high‑throughput screening of chromosomal abnormalities in future applications.

## Supplementary Material



## Data Availability

All data that support the findings of this study are included within the article. Source data for coffee-ring effect enable correlative super-resolution imaging of label-free chromosomes [23]. Supplementary material is available online [24].
