# Supplementary material for: Coffee-ring effect enables correlative super-resolution imaging of label-free chromosomes
Source: J R Soc Interface. 2026 Oct 14;23(243):20260072. doi: 10.1098/rsif.2026.0072 (PMC13682569; doi:10.1098/rsif.2026.0072)
Supplement: Supplementary file 1 [file rsif.2026.0072.zip › rsif.2026.0072/rsif20260072_si_001.docx]

Supplementary Materials for

**Coffee-ring effect enables correlative** **super-resolution imaging of label-free chromosomes**

Yanxin Dou^1,2,3,4*^, Valerie Swee Ting Goh^2,4^, Thomas Osipowicz^3*^, Christelle En Lin Chua^2*^

^1^ *Institute of Modern Physics, Chinese Academy of Sciences, Lanzhou 730000, China*

^2^ *Singapore Nuclear Research and Safety Institute, National University of Singapore, Singapore 118415, Singapore*

^3^ *Centre for Ion Beam Applications, Department of Physics, National University of Singapore,*

*Singapore 117542, Singapore*

^4^ *These authors contributed equally: Yanxin Dou, Valerie Swee Ting Goh*

** E-mail:* [*douyanxin@impcas.ac.cn*](mailto:douyanxin@impcas.ac.cn)*,* [*phyto@nus.edu.sg*](mailto:phyto@nus.edu.sg)*,* [*snrccel@nus.edu.sg*](mailto:snrccel@nus.edu.sg)

Supplementary Figures

Fig.S1 Typical resolution measurements (resolution <200 nm) of the nuclear microscopy using a grid for the chromosome imaging.

Fig.S2 Nuclear microscopy image of a small structure in a carbon film sample, showing a resolution of less than 200 nm.

Fig.S3 Nuclear microscopy (A) and optical microscopy (B) images of chromosomes. Small particles (size < 200 nm) can be identified by the nuclear microscope.

Fig.S4 TEM images of Chinese Hamster Ovary (CHO) chromosomes. Scale bar is 5 µm.

Fig.S5 TEM images of normal and dicentric chromosomes. (A,B) Dicentric chromosomes. (C,D) Overlap chromosomes.
